# Supplementary material for: End Sequence Analysis Toolkit (ESAT) expands the extractable information from single-cell RNA-seq data
Source: Genome Res. 2016 Oct;26(10):1397–410. doi: 10.1101/gr.207902.116 (PMC5052061; doi:10.1101/gr.207902.116)
Supplement: Supplemental Material [file supp_gr.207902.116_Supp_Materials.tar.gz › ESAT-esat_v0.1/doc/html/functions_vars_c.html]

ESAT: Class Members - Variables


ESAT


- Main Page
- Related Pages
- Packages
- Classes
- Files

- Class List
- Class Index
- Class Hierarchy
- Class Members

- All
- Functions
- Variables
- Enumerator

- a
- b
- c
- d
- e
- f
- g
- h
- i
- k
- l
- m
- n
- o
- p
- q
- r
- s
- t
- u
- v
- w


All Classes Namespaces Files Functions Variables Enumerator Pages

### - c -

- C\_CODE
  : broad.core.multiplealignment.MultipleAlignment
- CLIP\_AT\_BOUNDARIES
  : broad.pda.seq.rap.PlotAggregateRegions
- codonToAminoacid
  : broad.core.multiplealignment.AlignmentExtractor
- CONTINUOUS\_EXTENSION
  : broad.pda.seq.rap.CollectAnnotationEnrichments
- CONTROL
  : broad.pda.seq.rap.BuildRatioNullDistribution
  , broad.pda.seq.rap.GenomeScoringProgram
  , broad.pda.seq.rap.RatioPermutationPeakCaller
  , broad.pda.seq.rap.rna.TranscriptomeScoringProgram
- controlSamples
  : broad.pda.seq.protection.MultiSampleScanPeakCaller
- COORD\_SPACE
  : broad.pda.seq.rap.GenomeCommandLineProgram
- coordinateSpace
  : broad.pda.seq.rap.GenomeCommandLineProgram
  , umms.core.annotation.AnnotationList< T extends Annotation >
  , umms.core.model.AlignmentModel
  , umms.core.model.JCSAlignmentModel
- cs
  : umms.core.coordinatesystem.ShuffledIterator< T extends Annotation >
- ctrl
  : broad.pda.seq.protection.WindowScores
- ctrlModel
  : umms.core.model.score.MultiScore
- currentBase
  : broad.core.sequence.AbstractFastaParser
- CUTOFF
  : broad.pda.seq.rap.RatioPermutationPeakCaller
  , broad.pda.seq.rap.SkellamKLPeakCaller


---

Generated on Fri Aug 15 2014 15:11:17 for ESAT by  

 1.8.7
